# Outcomes after contact and distance elbow arthrodesis: a retrospective cohort study

**DOI:** 10.1186/s12891-026-10026-5

**Published:** 2026-05-27

**Authors:** Christian Fischer, M. Hückstädt, P Kobbe, T. Mendel, J. Eschweiler, S. Langwald, P. Schenk

**Affiliations:** 1 Department of Trauma and Reconstructive Surgery, BG Klinikum Bergmannstrost Halle gGmbH, Merseburger Straße 165, Halle (Saale), 06120 Germany; 2 Department of Trauma, Hand and Reconstructive Surgery, Martin Luther University Halle- Wittenberg, University Hospital Halle, Ernst-Grube-Straße 40, Halle (Saale), 06120 Germany; 3 Innovation Hub Musculoskeletal Surgery Halle (IHMS), Merseburger Straße 52, Halle (Saale), 06110 Germany

**Keywords:** Elbow arthrodesis, Salvage procedure, Plate fixation, Contact arthrodesis, Distance (defect) arthrodesis, Complex elbow trauma

## Abstract

**Background:**

Elbow arthrodesis (EA) is a rare salvage procedure for non-reconstructible elbow conditions and failed total elbow arthroplasty (TEA).

**Materials and methods:**

We retrospectively analyzed 20 adults treated with EA between 2010 and 2024, comparing contact arthrodesis (*n* = 12) with distance/defect arthrodesis after induced membrane technique (IMT) reconstruction (*n* = 8). Indications were infection in nine patients, trauma in three patients, osteoarthritis in three patients, non-union in three patients, and inflammatory arthritis in two patients. Outcomes included grip strength, wrist range of motion (ROM), patient-reported outcome measures (PROMS) Oxford Elbow Score (OES), Mayo Elbow Performance Score (MEPS), abbreviated Disabilities of the Arm, Shoulder and Hand (QuickDASH) and Single Assessment Numeric Evaluation (SANE), complications, and return to work.

**Results:**

Primary radiographic fusion was observed in 18 of 20 patients; two patients required revision surgery for nonunion, here secondary fusion was achieved with distance arthrodesis. The mean fixation angle was 102 ± 11°. At a mean follow-up of 4 ± 4 years, no statistically significant differences in PROMS or objective measures were detected between contact and distance arthrodesis. Relative grip strength on the operated side was 0.74 of the contralateral side, and wrist ROM was reduced. In dominant-hand injuries, fixation angle correlated with relative wrist radial-ulnar deviation ROM (r = 0.668, p = 0.009). Peri-implant fractures of the proximal ulna occurred in seven patients and intraoperative bleeding in four. Among patients of working age, 12 of 15 returned to work.

**Conclusions:**

No statistically significant differences in clinical outcomes were detected between defect/distance and contact arthrodesis; however, equivalence cannot be concluded from this underpowered exploratory cohort. Elbow arthrodesis remains a salvage procedure with substantial complication risk. Individualized fusion angle selection, local bone stock, infection status, and patient-specific occupational demands should be considered carefully during preoperative counseling and surgical planning.

**Level of evidence:**

Level III, retrospective comparative cohort study.

## Introduction

Elbow arthrodesis (EA) is a rare but important salvage procedure for patients with nonreconstructible elbow injuries, infections, arthritis or instability, and failed total elbow arthroplasty (TEA) [1, 2]. In these settings, the goal of EA is to achieve a pain-free, stable, and resilient upper extremity that, despite the loss of elbow mobility, allows functional use of the hand through compensation via the shoulder and wrist [3–5]. In selected patients after combat-related injuries of the upper extremity, arthrodesis has also been used as a limb-salvage alternative to amputation, to preserve a functional hand despite elbow or forearm injury [6].

The available evidence is largely limited to case series and small retrospective cohorts, typically comprising fewer than 20 patients [5, 7, 8]. This limited evidence base reflects both the rarity of EA and the complexity of the underlying salvage indications [6, 7, 9]. EA can be done by internal fixation with plates or intramedullary devices and external fixation, including circular frames in the presence of bone loss or infection. Medially positioned plating strategies and circular (ring) external fixation have been advocated as potential means to enhance fixation in compromised bone; however, the supporting evidence is limited [7, 8, 10]. Despite these proposed refinements, clinically relevant complications - including nonunion, persistent or recurrent infection, ulna fractures, and hardware-related problems - are repeatedly reported across the available series, underscoring the substantial technical and biological challenges inherent to elbow fusion [5, 7, 9, 11].

Functional outcome after EA depends not only on achieving a stable bony fusion, but also on the chosen fusion angle and the compensatory capacity of the shoulder and hand joints. Experimental and clinical studies show that fusion at a certain angle cannot preserve all activities of daily living, although there is no consensus on the optimal angle for different patient requirements [2, 5, 7, 12]. Existing case series suggest that patients adapt sufficiently for basic self-care and selected occupational tasks. In practice, heavy physical work and activities which require a larger range of motion (ROM) are rarely feasible after EA [2, 5, 7].

Patient satisfaction and functional adaptation after EA have been evaluated inconsistently across studies, with outcome assessment, when reported, most commonly relying on: DASH (Disabilities of the Arm, Shoulder and Hand) or QuickDASH scores, the Mayo Elbow Performance Score, generic pain scales, and return-to-work status [2, 5]. Heterogeneity of outcomes and varying follow-up periods continue to make it difficult for direct comparisons and derive clear expectations for patients and surgeons [2, 5, 7].

The aim of this study is to evaluate patients after EA by reporting indications, fusion rates, complications, and patient-reported functional outcomes after complex post-traumatic conditions or failed TEA. Furthermore, we assess differences between contact and distance arthrodesis as an explanatory analysis.

## Methods

This retrospective cohort study based on existing clinical records was conducted following approval by the responsible ethics committee and in compliance with the ethical principles of the Declaration of Helsinki for medical research in its most recent form (Ärztekammer Sachsen-Anhalt; approval No. 2025 − 242). All patients provided informed consent prior to participation in the study and data collection. This study is reported in accordance with the STROBE statement for observational studies.

A total of 20 patients who underwent EA between 2010 and 2024 were included. Patients were eligible for inclusion if they were ≥ 18 years of age and had an indication for arthrodesis due to trauma, fracture-related infection (FRI), periprosthetic joint infection (PJI), arthritis, or non-union of the elbow, with a minimum follow-up of at least one year. No tumor-related indications for EA occurred in this cohort. Recorded biometric data included sex, age, height, weight, and body mass index (BMI). Further clinical variables comprised the length of postoperative hospital stay (from surgery to discharge) and total in-hospital stay (from admission to discharge) in days; comorbidity and frailty scores (Charlson Comorbidity Index,:CCI; 5-item Modified Frailty Index, mFI-5); American Society of Anesthesiologists (ASA) physical status; and the operated side (left/right) were recorded, including whether it represented the dominant arm. Initial injuries that ultimately resulted in EA were classified according to the AO/OTA fracture classification.

Elbow arthrodesis was considered only as a salvage procedure after failure or infeasibility of reconstructive options. Indications reflected severe post-traumatic or post-revision pathology, including multiply operated injuries of the distal humerus and/or proximal ulna, periprosthetic infection after TEA with repeated revision surgery, fracture-related infection, non-union, and segmental bone defects after trauma. Most patients had not been treated primarily at our institution but were referred secondarily for definitive salvage reconstruction. The decision to perform contact or distance arthrodesis was based on the local bone stock, defect size, soft-tissue condition, infection status, and the feasibility of achieving stable compression. Contact arthrodesis was selected when sufficient bone apposition was possible, whereas distance/defect arthrodesis after induced membrane reconstruction was selected when segmental bone loss required intercalary reconstruction.

Infection-related indications were recorded descriptively as periprosthetic joint infection (PJI) or fracture-related infection (FRI). Because the primary aim of the study was functional outcome after completed arthrodesis rather than infectious disease outcome, and because the infection subgroup was small, formal inferential subgroup comparison between PJI and FRI was not performed.

Perioperative process parameters included operative time (minutes) and intraoperative fluoroscopy time (seconds). We recorded whether EA was performed as primary contact arthrodesis or as defect (distance) arthrodesis following stage II of the induced membrane technique. In defect arthrodesis cases, segmental bone loss was reconstructed using a tricortical iliac crest bone block combined with autologous iliac crest cancellous bone graft (bone-block technique previously published by our group). We further recorded whether fixation was achieved using a single-plate or double-plate construct and whether the radial head was resected.

The operative strategy, including one-stage versus two-stage arthrodesis, was determined by the local bone quality, soft-tissue and the presence or absence of persistent infection. Both contact and distance/defect arthrodesis could therefore be performed either as a single-stage or staged procedure. In contact arthrodesis, the goal was direct fusion of the prepared resection surfaces under compression. Distance/defect arthrodesis was performed as part of induced membrane technique (IMT) [13, 14]; in this cohort distance/defect arthrodesis was done by press-fit interposition of a bi- or tricortical iliac crest bone graft combined with autologous cancellous bone graft from the iliac crest. Fixation was performed using either a single locking plate or an angular-stable double-plate construct, depending on the primary stability achieved intraoperatively. The fusion angle was selected individually according to patient demands, hand dominance, hygiene requirements, and intraoperative feasibility of stable fixation.

No routine immobilization of the adjacent shoulder, wrist, or hand joints was performed. Postoperative loading of the operated extremity was generally restricted to a maximum of 5 kg as a precautionary measure, but rehabilitation and loading progression were individualized, and no dogmatic standardized aftercare protocol was used; this was added as a limitation of the study.

Bone consolidation was followed with serial plain radiographs during follow-up. CT was obtained only when consolidation could not be determined reliably on radiographs. Radiographs and available CT scans were independently assessed by two orthopaedic trauma surgeons and one radiologist. Bridging was evaluated at the proximal and distal docking interfaces (native bone-reconstruction segment/nonunion gap). On multiplanar CT reconstructions, cortical continuity was assessed for the anterior, posterior, medial, and lateral cortices; a cortex was considered bridged when uninterrupted cortical and/or trabecular continuity across the interface was visually evident. Consolidation was categorized as primary complete consolidation (≥ 3/4 cortices bridged at both interfaces without reoperation), partial consolidation (< 3/4 cortices bridged at either interface without reoperation), or secondary consolidation (complete consolidation achieved only after additional surgery, e.g., bone grafting or revision fixation).

Computed tomography (CT) was not performed routinely. CT was obtained only in cases in which osseous fusion could not be verified with sufficient confidence on standard projection radiographs, or when delayed union or non-union at the host-graft interfaces had to be excluded.

Hand grip strength was measured bilaterally (affected and contralateral limb) using a hand grip dynamometer (Seahan Industries, Eschborn, Germany). Wrist range of motion (flexion/extension and radial-ulnar deviation) was measured using a goniometer and documented using the standardized measurement form of the German Social Accident Insurance (Deutsche Gesetzliche Unfallversicherung, DGUV; form F4222). Patient-reported outcomes were assessed using validated instruments: Oxford Elbow Score (OES; 0–48, higher scores indicate better outcome), Mayo Elbow Performance Score (MEPS; 0–100, higher scores indicate better outcome), abbreviated Disabilities of the Arm, Shoulder and Hand (QuickDASH; 0–100, higher scores indicate greater disability), and Single Assessment Numeric Evaluation (SANE; 0–100, higher scores indicate better function). Return to work was recorded at follow-up as yes/no and was defined as resumption of paid employment in the same job or with minor task modifications; time to return to work and reasons for non-return were not systematically captured.

Wrist ROM was measured clinically with a goniometer; formal inter-rater reliability testing for goniometric wrist measurements was not performed. Standardized functional task simulation (e.g., hand-to-mouth or hand-to-back tasks) and a dedicated satisfaction questionnaire were not part of the retrospective assessment; satisfaction was therefore only indirectly reflected by SANE and PROM results.

Standardized preoperative PROMs could not be collected routinely before arthrodesis, because all patients were referred secondarily to our institution after previous external surgical treatment. Moreover, the severity of the initial injuries and the high number of previous operations would likely have confounded preoperative PROM assessment. Therefore, PROMs are reported as follow-up outcomes only, and the lack of baseline PROMs was treated as a study limitation.

Differences in the distribution of categorical or dichotomous variables between patients with contact or distance arthrodesis were analyzed using the Chi-square and Fisher’s Exact Test. Differences in continuous variables, such as the Charlson Comorbidity Index (CCI), modified Frailty Index (mFI-5), and ASA score, were assessed using the Mann-Whitney U-test after checking for normal distribution using the KS-test. Differences in metric variables were examined using a multivariate general linear model (GLM). Interobserver agreement for radiographic bony fusion among the three reviewers was evaluated using kappa statistics. The correlation between the fixation angle of the elbow and functional outcomes was analyzed using bivariate correlation, only in patients with injury to the dominant hand. For categorical variables, frequencies and corresponding percentages were reported. For metric variables, the mean and standard deviation were provided, along with the range (minimum to maximum). The statistical analysis was performed using SPSS Statistics (V. 29; IBM Corp, Armonk, NY) with a p-value of < 0.05 considered as statistically significant.

Exploratory sensitivity analyses were added for robustness. These included descriptive reassessment of group comparisons after excluding the two cases that required secondary consolidation and non-parametric reassessment of the association between fixation angle and functional outcomes in dominant-side injuries. Because of the small cohort size, these sensitivity analyses were considered supportive and hypothesis-generating.

## Results

**Fig. 1:**
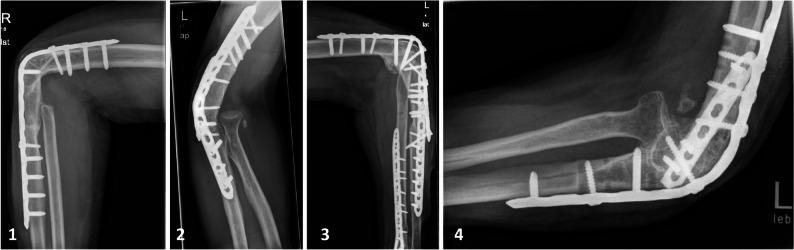
Elbow arthrodesis techniques: distance (defect) arthrodesis with a customized single locking plate construct (1, 2) and contact arthrodesis with a customized double locking plate construct (3, 4)

The study cohort comprised 20 patients (12 contact-, 8 distance arthrodesis), including 13 men and seven women (*p* = 0.158), with a mean age of 52 ± 17 years (range 23–90 years, *p* = 0.443). Mean BMI was 25 ± 4 kg/m² (range 17–31, *p* = 0.412). The two EA techniques applied in this cohort are depicted in Fig. 1. No difference was found between patients with contact arthrodesis or distance arthrodesis in terms of the side of the dominant hand (*p* = 0.642) and operated side (*p* = 0.670).

According to the AO fracture classification, five fractures were classified as type A, six as type B, and nine as type C (*p* = 1.000). The most common indication for surgery was infection, including PJI or FRI, in nine of 20 patients. Trauma, osteoarthritis, and non-union were each observed in three of 20 patients, while inflammatory arthritides were present in two of 20 patients. Among the nine infection-related cases, causative organisms included Staphylococcus aureus or Staphylococcus epidermidis. Because of the small number of PJI/FRI cases and the functional focus of the study, infection phenotype was analyzed descriptively only. Pre-existing nerve injury was documented in four of 20 patients, whereas no neurological deficits were present in the remaining patients. No differences between patients with contact or distance arthrodesis was found for CCI (*p* = 0.416), mFI5 (*p* = 0.195) and ASA (*p* = 0.403). Regarding comorbidity, ten of 20 patients had a Charlson Comorbidity Index (CCI) = 0. The remaining patients had CCI values ranging from 2 to 6. According to the modified five-item frailty index (mFI-5), 14 of 20 patients had a score of 0, while six patients had scores from 1, 2 or 3. The distribution of ASA scores in the study population showed a predominance of ASA status 2 (*n* = 9), followed by ASA status 3 (*n* = 6), ASA status 1 (*n* = 3), and ASA status 4 (*n* = 2), without significant different distribution between patients with contact and distance arthrodesis (*p* = 0.538). Mean operative time was 144 ± 44 min (range 70–236 min, *p* = 0.935). The mean intraoperative fluoroscopy time was 12 ± 10 s (range 3–35 s) and differed between patients with contact (16 ± 11s) and distance arthrodesis (7 ± 2s, *p* = 0.033). Table 1 shows whether single or double plates were used and whether a radial head reconstruction was performed.

**Table 1:** Radial head resection by elbow arthrodesis type (contact- and distance arthrodesis, (AD))

		Contact AD	Distance AD	*P*
AD implant	Single plate	6	1	0.151
	Double plate	6	7	
Radial head resection	yes	5/12	4/8	1.000

The angle of fixation was 102 ± 11° (range: 70–119°, *p* = 0.461). The mean postoperative hospital stay was 10 ± 4 days (range 5–19 days, *p* = 0.691), while the total length of hospital stay averaged 12 ± 4 days (range 6–20 days, *p* = 0.699). Intraoperative or perioperative surgical complications occurred in six patients (*p* = 1.000). These included intraoperative bleeding in four cases, as well as one case of nerve injury and one early postoperative infection. During follow-up, peri-implant fractures were observed in 7 patients. In addition, infection and non-union occurred in two patients each, and two patients reported chronic pain.

Peri-implant ulnar fractures were managed with revision osteosynthesis using single- or double-plate fixation according to fracture morphology and residual bone stock. Recurrent infection and non-union were treated with staged revision concepts. In both non-union cases, secondary osseous fusion was achieved after revision surgery with additional cancellous bone grafting from the iliac crest.

The average time until follow-up examination was 4 ± 4 years (range: 1–14 years, *p* = 0.533). At FU no significant differences were found for the functional outcome, comparing contact and distance arthrodesis (Table 2, *p* > 0.108). Primary bony fusion was achieved in 18 of 20 patients. In two cases treated with distance arthrodesis, secondary fusion after additional surgery (cancellous bone grafting) was observed. CT was obtained only in selected cases in which fusion was not sufficiently assessable on projection radiographs; therefore, a routine radiograph-versus-CT concordance analysis was not possible. Interobserver agreement for the categorical plain radiograph and/or CT-based consolidation assessment was high (weighted κ = 0.92, *p* < 0.001).

**Table 2:** Functional outcomes at follow up of patients with contact and distance elbow arthrodesis, presented as mean and standard deviation for the entire cohort and p value for differences between both arthrodesis groups. Handgrip strength is reported in kg, wrist range of motion (ROM) in degrees (°), and relative values as ratios of the injured to the uninjured side. OES: Oxford Elbow Score; MEPS: Mayo Elbow Performance Score; SANE: Single Assessment Numeric Evaluation; QuickDASH: abbreviated Disabilities of the Arm, Shoulder and Hand; Rel.: relative; AD: arthrodesis

	Total	Contact AD	Distance AD	*p*
Handgrip injured (kg)	29 ± 12	28 ± 13	29 ± 10	0.862
Handgrip uninjured (kg)	38 ± 11	36 ± 11	41 ± 10	0.330
Rel. handgrip	0.74 ± 0.20	0.74 ± 0.18	0.75 ± 0.24	0.982
OES	28 ± 5	28 ± 4	28 ± 7	0.892
MEPS	51 ± 9	50 ± 9	53 ± 10	0.460
SANE	63 ± 16	66 ± 11	57 ± 22	0.218
QuickDASH	43 ± 10	46 ± 9	39 ± 11	0.108
Wrist flexion-extension ROM uninjured (°)	101 ± 14	99 ± 16	104 ± 13	0.446
Wrist flexion-extension ROM injured (°)	58 ± 9	56 ± 8	59 ± 10	0.460
Wrist radial-ulnar deviation ROM uninjured (°)	80 ± 13	78 ± 15	81 ± 11	0.648
Wrist radial-ulnar deviation ROM injured (°)	49 ± 10	49 ± 10	48 ± 10	0.891
Rel. wrist flexion-extension ROM	0.79 ± 0.12	0.80 ± 0.14	0.78 ± 0.10	0.764
Rel. wrist radial-ulnar deviation ROM	0.85 ± 0.15	0.87 ± 0.15	0.82 ± 0.14	0.424

The exploratory sensitivity analysis excluding the two patients who required secondary consolidation did not materially change the interpretation that no statistically significant differences in functional PROMs or objective functional measures were detected between patients with contact and distance arthrodesis among patients with achieved fusion. This analysis does not establish equivalence between techniques. Non-parametric reassessment of the dominant-side angle analysis showed the same direction of association between fixation angle and relative wrist radial-ulnar deviation ROM. These analyses were interpreted cautiously because of the very small subgroup sizes.

In patients with injury to the dominant hand (*n* = 14), a significant bivariate correlation was observed between the fixated elbow angle and the relative wrist radial-ulnar deviation ROM, with a correlation coefficient of *r* = 0.668 (*p* = 0.009). No significant correlations with the fixation angle in the elbow were found for any other functional outcome measures at the time of follow-up. Because this exploratory correlation analysis was not adjusted for multiple comparisons across the assessed outcome variables, the finding should be interpreted as hypothesis-generating. (Table 3)

**Table 3:** Bivariate correlations between the fixation angle in the elbow and functional outcome measures at follow-up in the dominant-side injury subgroup. Correlation coefficients (r), if significant, and p-values are reported. Handgrip strength is reported in kg, wrist ROM in degrees (°), and relative values as ratios of the injured to the uninjured side. OES: Oxford Elbow Score; MEPS: Mayo Elbow Performance Score; SANE: Single Assessment Numeric Evaluation; QuickDASH: abbreviated Disabilities of the Arm, Shoulder and Hand; ROM: range of motion; Rel.: relative

	*r*	*p*
Handgrip injured (kg)	-	0.448
Rel. handgrip	-	0.309
OES	-	0.895
MEPS	-	0.433
SANE	-	0.200
QuickDASH	-	0.863
Wrist flexion-extension ROM injured (°)	-	0.916
Wrist radial-ulnar deviation ROM injured (°)	-	0.240
Rel. wrist flexion-extension ROM	-	0.615
Rel. wrist radial-ulnar deviation ROM	0.668	0.009

Five patients were retired at the time of treatment and were therefore excluded from the return-to-work analysis. Of the remaining 15 patients, 12 of 15 were able to return to work, whereas three of 15 patients did not resume occupational activity during the follow-up period (*p* = 1.000). Among patients who returned to their previous occupation, available occupational descriptions indicated predominantly desk-based work rather than heavy manual labor.

Detailed time to return to work, exact workload categories, job modifications, retraining pathways, functional limitations after return, and reasons for non-return were not documented systematically; therefore, return-to-work results should be interpreted as a binary occupational participation endpoint rather than as a detailed work-capacity analysis.

### Study strengths, limitations, future directions and clinical implications

This study has several strengths, including a clearly defined rare salvage cohort, standardized radiographic consolidation criteria, independent radiographic assessment, use of established PROMs, and comparatively long follow-up for this uncommon procedure. Although the cohort is small in absolute terms, a series of 20 patients represents a relatively large single-center experience for elbow arthrodesis in the available literature.

Important limitations are the retrospective design, clinical heterogeneity, potential referral and selection bias, confounding by indication between contact and distance arthrodesis, limited availability of standardized baseline PROMs, selective rather than routine CT use, and incomplete occupational and rehabilitation details. The heterogeneous follow-up duration (range 1–14 years) may also introduce time-at-risk bias, because late mechanical failures can only be observed in patients with sufficiently long follow-up. Future studies should use prospective multicenter registries, standardized preoperative and longitudinal PROM assessment, predefined radiographic follow-up protocols including CT criteria, detailed rehabilitation documentation, longer follow-up, and structured return-to-work metrics. Clinically, the results support elbow arthrodesis as an individualized salvage procedure aimed at pain reduction and mechanical stability rather than restoration of normal elbow function.

## Discussion

The present study provides additional clinical data of 20 patients with distance or contact EA after complex post-traumatic conditions and failed TEA, and represents one of the larger single-center cohorts reported to date. Our results confirm that EA can achieve a high rate of bone fusion and allows functional use of the upper extremity. However, because of the small sample size, heterogeneous indications, and confounding by indication, the absence of statistically significant differences between contact and distance arthrodesis must not be interpreted as proof of equivalence.

### Radiographic fusion and structural phenotype

In our cohort, radiographic fusion was achieved in all patients with contact arthrodesis and almost each patient with distance arthrodesis, comparable to union rates reported in small salvage series. Koller et al. (mixed salvage indications, largely post-traumatic) reported solid union in 14 of 14 elbows at a mean follow-up of 62 months [5]. Sheean et al. (combat-related trauma) achieved fusion in 5 of 5 patients with confirmed fusion after a mean of 714 days from arthrodesis [6]. Sala et al. (post-traumatic, Ilizarov fixation) reported union in 3 of 4 patients at a mean follow-up of 23 weeks [8]. By contrast, Otto et al. (infected failed TEA) reported no confirmed bony union in 0 of 5 elbows at final follow-up, with all five patients undergoing reoperation [4]. However, these studies did not differentiate between distance and contact arthrodesis. In the present study, CT was used selectively only when radiographic fusion was uncertain, which strengthens assessment in equivocal cases but prevents routine modality-specific concordance analysis. Follow-up examinations after a long period of time still reveal patients who show no fusion, as our results also demonstrate.

### Complications and reoperations

Complications mostly included peri-implant ulnar fractures, infection, and non-union, underscoring the technical and biological challenges associated with elbow fusion, particularly in patients with compromised bone quality or infection-related indications [1–5, 8]. Peri-implant fractures were treated by revision osteosynthesis using single- or double-plate constructs, whereas recurrent infection and non-union were managed by staged revision concepts. These findings highlight that EA remains a demanding salvage procedure, even in specialized centers. In our cohort, no significant differences in fusion rates, complication rates, or patient reported outcome measures (PROMs) were observed between patients with contact and distance arthrodesis. This finding should be interpreted as absence of evidence for a difference in this underpowered cohort, not as evidence of equivalence. The significantly longer fluoroscopy time observed in contact arthrodesis should not be overestimated, as the absolute difference was only a few seconds and is unlikely to be clinically relevant for radiation exposure or infection risk.

EA is generally reserved for end-stage, non-reconstructable elbow pathology [2, 4, 5]. Contemporary reviews consistently characterize it as a salvage procedure with narrowly defined indications, deployed when revision fixation, biological reconstruction, interposition arthroplasty, or TEA are not feasible or carry an unacceptably high risk of failure. However, our results are based on the analysis of twenty patients, which represents a relatively large cohort for EA but remains limited from a statistical perspective. Therefore, our findings should be interpreted with caution and not as confirmation of a single superior technique, particularly given the absence of relevant differences between the compared approaches. Instead, our data suggest that, across heterogeneous indications, satisfactory clinical outcomes can be achieved when the essential prerequisites for fusion are met.

Concurrently, the literature describes particularly poor results when EA is performed as salvage after an infected failed TEA, although the available evidence is limited to small case series [3, 4, 8]. In this setting, severe bone loss, soft-tissue compromise, and multiple prior operations limit stable compression and local healing capacity, reducing union rates and functional recovery [4, 5]. In our cohort, infection-related indications comprised PJI and FRI, with Staphylococcus aureus or Staphylococcus epidermidis being the main pathogens detected. We agree that PJI and FRI represent biologically different entities; however, the present study was designed to assess functional outcome after arthrodesis, not infection-eradication endpoints, and the infection subgroup was too small for a meaningful inferential PJI-versus-FRI comparison. Evidence on prognostic factors for EA is scarce; most insights on infection, bone loss, and soft-tissue compromise come from the TEA literature, particularly revision and infection-failure studies [3, 4, 11]. These data indicate that outcomes after elbow AD are context-dependent; residual bone stock, soft-tissue status, and surgical history should inform preoperative risk stratification, counseling, and functional expectation management. Our cohort included both PJI and FRI, and our outcomes support an important conceptual distinction: infection is not a uniform prognostic variable; rather, the relevant determinants are the reconstructability of bone and soft tissue and the feasibility of achieving stable compressive fixation. Adequate debridement and infection control are mandatory in this context. This is in line with current conceptual discussions that caution against overgeneralization and instead emphasize indication based on local biology and mechanical conditions [2, 5, 7].

A frequently cited concern regarding defect (“distance”) AD is that segmental bone loss reduces the available apposition surface, compromises local biology, and complicates fixation, thereby increasing the risk of nonunion. Accordingly, technical approaches have focused on enlarging the fusion surface and enhancing construct stability, for example through shaping osteotomies, compression-based fixation, and staged reconstruction strategies in biologically unfavorable conditions [15]. Our findings refine this concept in two respects. First, the clustering of nonunions in the defect group is consistent with the broader notion that extensive bone loss and impaired vascularity increase the risk of impaired healing and may necessitate systematic host optimization (e.g., smoking cessation, management of relevant comorbidities) as well as standardized staged protocols. Second, among patients who achieved union, defect arthrodesis was not associated with a consistent functional disadvantage compared with contact arthrodesis. This suggests that the “distance” phenotype primarily affects the pathway to osseous consolidation, rather than inevitably leading to inferior long-term function, provided that a stable and pain-limited lever arm can be restored [16, 17]. Functional status after EA is largely determined by the selected fusion position and compensatory motion at adjacent joints. Experimental analyses and clinical observations indicate that fusion in a functional position results in relevant activity limitations; however, shoulder and wrist compensation can partially mitigate these constraints [18, 19].

There is no consensus on the optimal plating strategy for EA, and recommendations rely on limited comparative evidence. Although higher construct stiffness (e.g., double plating) is often assumed to be beneficial, clinical support is sparse. Biomechanical data suggest that different configurations can provide similar overall stability but differ in failure modes and stress distribution, and that variables such as working length and screw density may be as important as plate number [20–22].

Accordingly, our cohort was not designed to compare fixation techniques but to assess outcome differences between contact and defect arthrodesis. Plate count therefore represents an incomplete surrogate for key mechanical determinants, including achievable compression and stress concentration at construct transitions [21, 23]. Peri-implant ulnar fractures are relevant in this context, as comparable late fractures in other settings are frequently linked to stress-riser effects at implant termini and often require reoperation. Their occurrence after consolidated arthrodesis suggests persistent long-term vulnerability of the ulna, which should be considered in construct planning and counseling [24–26].

### Functional outcome, fusion angle, and work participation

Fusion angle selection remains one of the key and most debated decisions in EA. Functional studies addressing activities of daily living indicate that a flexion position around 90° represents a practical compromise, whereas clinical series and reviews emphasize that no single angle is optimal for all patients and recommend individualization based on occupation, hygiene requirements, and preoperative task simulation [25, 26]. In our cohort, fixation angle was significantly associated with relative wrist radial-ulnar deviation range of motion in dominant-side injuries. This is consistent with the concept that fusion position can influence distal compensatory kinematics and the extent to which the wrist contributes to placing the hand in space. However, this exploratory analysis was based on a small subgroup and was not corrected for multiple comparisons. Prior work on elbow stiffness similarly highlights compensation through the shoulder and wrist as a major determinant of functional arm use [5, 26]. This finding supports an approach already advocated in the literature: preoperative simulation of relevant tasks and shared decision-making to align the fusion angle with patient-specific priorities.

Among working-age patients, 12 of 15 returned to work, indicating that EA can provide sufficient stability for occupational participation in selected patients. Available occupational information indicated mainly desk-based work among those who resumed their previous occupation; therefore, these findings cannot be generalized to heavy manual labor. PROMs nonetheless reflected persistent functional limitations, consistent with prior reports that arthrodesis enables compensatory limb use but does not restore normal upper-extremity function. Return to work likely depends on occupational demands, and work performance, time to return, and job modifications were not examined in detail in our study. Overall, these findings support EA as a salvage option prioritizing pain control and mechanical stability when TEA is unsuitable due to infection risk, bone loss, or high-demand use.

EA remains rare; accordingly, single-center series are typically small and clinically heterogeneous, which can limit external validity. The retrospective, non-randomized design introduces potential selection bias, including confounding by indication, as contact and defect arthrodesis reflect different levels of structural compromise. Individual factors such as indication subtype (notably infection phenotypes), prior surgical burden, and comorbidities may further affect outcomes. Baseline (pre-injury) function was not available, and variable follow-up durations may influence measured endpoints and the detection of late implant failure. In addition, binary radiographic classification of fusion is pragmatic but may not capture delayed union or clinically relevant fibrous union. Wrist ROM was measured by goniometry without formal inter-rater reliability testing, pain was not reported as a separate primary endpoint, functional task simulation was not available, and patient satisfaction was assessed only indirectly. These limitations highlight the value of multicenter studies with standardized definitions for indication phenotypes, fixation principles, fusion-angle rationale, complication classification (including ulnar fracture patterns), rehabilitation protocols, return-to-work metrics, and longitudinal PROM reporting.

## Conclusion

In this single-center cohort study, EA was associated with a high rate of radiographic fusion in cases where stable compression and sufficient local biology could be achieved. No statistically significant differences in clinical and patient-reported outcomes were detected between contact and defect/distance arthrodesis; however, these findings should not be interpreted as proof of equivalence because the comparison was underpowered and confounded by indication. Nonunion requiring secondary revision occurred only in the defect subgroup. Late mechanical complications—particularly peri-implant ulnar fractures—were observed and are relevant for preoperative counseling and construct planning. Fusion angle selection should be individualized; the observed association between fixation angle and wrist compensatory motion suggests that elbow position may influence distal compensatory mechanics. Nonetheless, interpretation of subgroup comparisons is limited by the rarity of the procedure and the heterogeneity of salvage indications. Accordingly, the subgroup comparison should be interpreted as exploratory rather than as evidence of equivalence or superiority of either technique.

## Data Availability

The de-identified data that support the findings of this study are available from the corresponding author upon reasonable request and subject to institutional approval and applicable data-protection regulations.
